# Toward the Identification of Extra-Oral TAS2R Agonists as Drug Agents for Muscle Relaxation Therapies via Bioinformatics-Aided Screening of Bitter Compounds in Traditional Chinese Medicine

**DOI:** 10.3389/fphys.2019.00861

**Published:** 2019-07-16

**Authors:** Mingzhi Luo, Kai Ni, Yang Jin, Zifan Yu, Linhong Deng

**Affiliations:** ^1^Changzhou Key Laboratory of Respiratory Medical Engineering, Institute of Biomedical Engineering and Health Sciences, Changzhou University, Changzhou, China; ^2^Bioengineering College, Chongqing University, Chongqing, China

**Keywords:** TAS2Rs, muscle relaxation, biomechanics, bitter compounds, TCM, drug screening, bioinformatics

## Abstract

Significant advances have been made in the past decade in mapping the distributions and the physiological functions of extra-oral bitter taste receptors (TAS2Rs) in non-gustatory tissues. In particular, it has been found that TAS2Rs are expressed in various muscle tissues and activation of TAS2Rs can lead to muscle cell relaxation, which suggests that TAS2Rs may be important new targets in muscle relaxation therapy for various muscle-related diseases. So far, however, there is a lack of potent extra-oral TAS2R agonists that can be used as novel drug agents in muscle relaxation therapies. Interestingly, traditional Chinese medicine (TCM) often characterizes a drug’s property in terms of five distinct flavors (bitter, sweet, sour, salty, and pungent) according to its taste and function, and commonly regards “bitterness” as an intrinsic property of “good medicine.” In addition, many bitter flavored TCM are known in practice to cause muscle relaxation after long term use, and in lab experiments the compounds identified from some bitter flavored TCM do activate TAS2Rs and thus relax muscle cells. Therefore, it is highly possible to discover very useful extra-oral TAS2R agonists for muscle relaxation therapies among the abundant bitter compounds used in bitter flavored TCM. With this perspective, we reviewed in literature the distribution of TAS2Rs in different muscle systems with a focus on the map of bitter flavored TCM which can regulate muscle contractility and related functional chemical components. We also reviewed the recently established databases of TCM chemical components and the bioinformatics software which can be used for high-throughput screening and data mining of the chemical components associated with bitter flavored TCM. All together, we aim to present a knowledge-based approach and technological platform for identification or discovery of extra-oral TAS2R agonists that can be used as novel drug agents for muscle relaxation therapies through screening and evaluation of chemical compounds used in bitter flavored TCM.

## Introduction

Humans can distinguish five basic taste modalities including bitter, sweet, sour, salty, and umami, classified by modern science, which forms the principal gustatory perception often experienced during food consumption (Drewnowski and Gomez-Carneros, 2000; Meyerhof, 2005). Interestingly, traditional Chinese medicine (TCM), developed over 1000 of years based on experience of fighting diseases, has also established a “Flavor Theory” that categorizes the medicinal materials into similar “five flavors” including bitter, sweet, sour, salty, and pungent, according to the material’s taste and function (Hesketh and Zhu, 1997; Lukman et al., 2007; Ma et al., 2013; Zhang et al., 2016; Dragos and Gilca, 2018). Table 1 shows in parallel the five tastes in gustatory perception and the five flavors in TCM, which are very similar by all means.

**TABLE 1:** Comparison of five tastes in gustatory perception and five flavors in TCM.

**Taste**	**Common food**	**Function substrate**	**Targets**	**Flavors**	**Common TCM**	**Function substrate**	**Meridians**
Sour	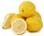 Lemon	Acid	Ion channels	Sour	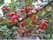 *Cornus officinalis* fruit	Organic acid Tannin	Liver Kidney
Salt	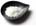 Salt	Salt chloride	Ion channel (ENaC)	Salt	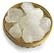 Mirabilite	Salt sulfate	Stomach Intestinal tract
Bitter	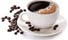 Coffee	Coffeine	TAS2R	Bitter	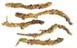 *Coptis chinensis* root	Alkaloid (Berberine) Flavone	Liver Lung Stomach Intestinal tract Heart
Sweet	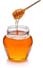 Honey	Sugar	TAS1R2 TAS1R3	Sweet	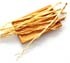 *Codonopsis pilosula* root	Polysaccharide Saponin	Lung Spleen
Umami	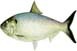 Fish	Amino acid (Glutamate)	TAS1R1 TAS1R3	Pungent	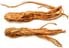 *Angelica sinensis* root	Volatile oil (Ligustilide) Amino acid Saponin	Heart Spleen Liver

It is also strongly believed and apparently supported by empirical evidences in TCM that “Good medicine tastes bitter” (Lu et al., 2017). In fact, even most of the modern medications also taste bitter. Conversely, it may also be true that bitter substance is good for medicine. According to a recent report, 38% of all the tested materials used for treating cardiovascular, respiratory and digestive diseases in TCM are of bitter flavors (Tang et al., 2018). To date, there has been a vast source of chemical compounds identified from bitter flavored TCM but their functional targets remain to be identified, which undoubtedly provides a gold mine for searching potential good drugs for treating various diseases (Editorial, 2007; Cheung, 2011).

Intriguingly, the type II taste receptors (TAS2Rs) that recognize bitter tastants have only been identified in 2000 (Adler et al., 2000; Chandrashekar et al., 2000). Since then, many studies have confirmed that TAS2Rs are expressed not only in taste buds but also in extra-oral tissues (Behrens and Meyerhof, 2013; Maina et al., 2018; Scadding, 2018) including heart, skeletal and smooth muscle (Shaik et al., 2016). Although bitter taste is initially assumed as a self-protection mechanism to prevent humans from ingesting toxins, it is now known that bitter taste can actually have far more roles to play than mere self-protection (Lee et al., 2019). For example, bitter taste substances such as quinine can cause airway smooth muscle relaxation, which may be useful for asthma treatment (Deshpande et al., 2010). Studies have also revealed that TAS2Rs mediate relaxation of smooth muscle in other organs such as bladder (Zhai et al., 2016), blood vessel (Manson et al., 2014), and uterus (Zheng et al., 2017). Therefore, TAS2Rs may be novel targets for screening and thus discovering new drug agents for muscle relaxation therapies to treat various diseases (Kim et al., 2018). In such pursuit, the existing databank of bitter flavored TCM and their correlated bitter components will be very useful as starting points (Sucher, 2013). On the other hand, the exploration of bitter flavored TCM for novel agonists that target TAS2Rs in extra-oral systems will inevitably contribute to modernizing TCM (Pieroni and Giusti, 2009; Su and Miller, 2015; Gotoh et al., 2018).

In this perspective, here, we reviewed the recent advances in the understanding about the structure, distribution and function of TAS2Rs in diverse muscle tissues, with highlights of the perceptive profile of TAS2Rs for bitter components from bitter flavored TCM. We also reviewed the bitter flavored TCM that are already used in treatment of muscle related symptoms in cardiovascular, respiratory, gastrointestinal, bladder, and uterus systems. Finally, we discussed the future opportunities of using *in silico* analysis to screen extra-oral TAS2R agonists as novel muscle relaxants from bitter flavored TCM.

## Structure and Function of TAS2Rs

### Structure of TAS2Rs

In humans, TAS2Rs are a family of 25 type A G protein coupled receptors (GPCRs) (versus 35 TAS2Rs in rats and mice) according to their structure and binding site location (Di Pizio et al., 2016, 2019; Alfonso-Prieto et al., 2019). Although TAS2Rs have been found in many species, in each species their genes are not highly conserved in terms of sequence. TAS2Rs contain 291–334 amino acids, share 23–86% sequence identity (Chandrashekar et al., 2000; Scadding, 2018), and cluster to chromosomes 5, 7, 12 in humans but 2, 3, 4, and 15, 2, 6 in rats and mice, respectively (Wu et al., 2005). Interesting is that 33 of 35 *TAS2R* genes in rats and mice exhibit a one-to-one homology (Table 2). Furthermore, humans, rats and mice also contain 16 orthologous *TAS2R* genes (Shi et al., 2003; Pearson, 2013).

**TABLE 2:** The orthologous genes in human, rat, and mouse (Foster et al., 2013).

**No.**	**Human**	**Rat**	**Mouse**
1	*TAS2R1*	*rTas2r119*	*mTas2r119*
2	*TAS2R3*	*rTas2r137*	*mTas2r137*
3	*TAS2R4*	*rTTas2r108*	*mTas2r108*
4	*TAS2R5*		
5	*TAS2R7*	*rTas2r130*	*mTas2r130*
6	*TAS2R8*		
7	*TAS2R9*		
8	*TAS2R10*	*rTas2r114*	*mTas2r114*
9	*TAS2R13*	*rTas2r121*	*mTas2r121*
10	*TAS2R14*	*rTas2r140*	*mTas2r140*
11	*TAS2R16*	*rTas2r118*	*mTas2r118*
12	*TAS2R19*		
13	*TAS2R20*		
14	*TAS2R30*		
15	*TAS2R31*	*rTas2r136*	*mTas2r136*
16	*TAS2R38*	*rTas2r138*	*mTas2r138*
17	*TAS2R39*	*rTas2r139*	*mTas2r139*
18	*TAS2R40*	*rTas2r144*	*mTas2r144*
19	*TAS2R41*	*rTas2r126*	*mTas2r126*
20	*TAS2R42*	*rTas2r145*	*mTas2r131*
21	*TAS2R43*		
22	*TAS2R45*		
23	*TAS2R46*	*rTas2r120*	*mTas2r120*
24	*TAS2R50*		
25	*TAS2R60*	*rTas2r135*	*mTas2r135*
		*rTas2r102*	*mTas2r102*
		*rTas2r103*	*mTas2r103*
		*rTas2r104*	*mTas2r104*
		*rTas2r105*	*mTas2r105*
		*rTas2r106*	*mTas2r106*
		*rTas2r107*	*mTas2r107*
		*rTas2r109*	*mTas2r109*
		*rTas2r110*	*mTas2r110*
		*rTas2r113*	*mTas2r113*
		*rTas2r116*	*mTas2r116*
		*rTas2r117*	*mTas2r117*
		*rTas2r123*	*mTas2r123*
		*rTas2r124*	*mTas2r124*
		*rTas2r125*	*mTas2r125*
		*rTas2r129*	*mTas2r129*
		*rTas2r134*	*mTas2r134*
		*mTas2r143*	*mTas2r143*
			*mTas2r115*
			*mTas2r122*
		*rTAS2R7l*	
		*rTAS2R13*	

*Based on the ortholog analysis of TAS2Rs in human, rat, and mouse (Wu et al., 2005).*

TAS2Rs consist of short extracellular N-terminus and intracellular C-terminus, seven transmembrane helices (TMs) connected by three extracellular loops (ECLs) and three intracellular loops (ICLs) (Zhang et al., 2017). TMs and ECLs contain binding sites where bitter tastants bind to and facilitate conformational changes of TAS2Rs toward an “active state,” which allows activation of TAS2Rs and downstream signaling (Bufe et al., 2002; Biarnés et al., 2010). ICLs play a major role in the interaction of TAS2Rs with G proteins (Brockhoff et al., 2010; Sanematsu et al., 2014). For instance, interaction at ICL3 may stabilize the inactive state of TAS2R16 while structural changes in the intracellular region are correlated with activation, as demonstrated by Chen et al. (2018) using molecular dynamics simulation.

### Canonical Signaling of TAS2Rs

Activation of TAS2Rs initiates Ca^2+^ signaling with a cascade reaction of G protein (α and βγ subunits), phospholipase C β2 (PLCβ2), and inositol trisphosphate (IP_3_). Upon receptor activation, the G protein dissociates α and βγ subunits. The latter activates PLCβ2, leading to a release of Ca^2+^ from IP_3_-sensitive Ca^2+^ stores. In addition, it has been found that the increase in intracellular free calcium concentration ([Ca^2+^]_i_) induced by TAS2R agonists is correlated with the expression level of the subtype of TAS2Rs (An and Liggett, 2018).

In taste cells, TAS2R signaling involves the coupling of G protein gustducin to PLCβ2 to induce Ca^2+^ release from IP_3_-sensitive Ca^2+^ stores, and Na^+^ influx through transient receptor potential ion channels melastatin 5 (TRPM5) channels, which depolarizes the cell and causes the paracrine release of neurotransmitter ATP through the gap junction channels. Ultimately, ATP activates purinergic receptors on nerve cells to initiate the perception of bitterness. In other cell types, however, TAS2Rs can optionally couple to several G proteins in a cell type-dependent manner, such as the coupling of TAS2Rs to G_iα1,2,3_ in human airway smooth muscle cells (SMCs; Chen et al., 2018).

### Binding Site of TAS2Rs

**FIGURE 1:**
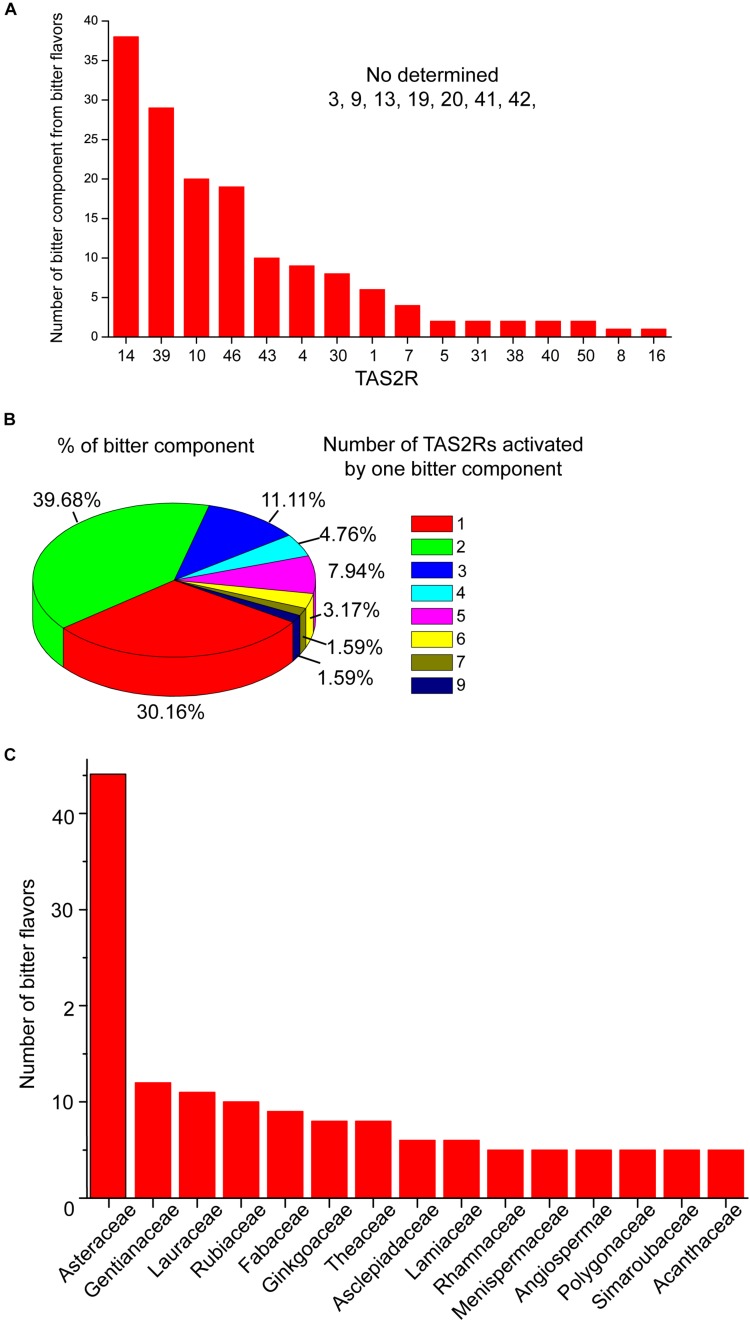
The activation profile of TAS2Rs by bitter components from bitter flavored TCM. **(A)** Bar graph shows the TAS2Rs that can be activated by different bitter components. **(B)** The profile of bitter components which can activate different number of TAS2Rs. **(C)** The family in which bitter components with specific TAS2R have been found. The primary data are shown in Supplementary Table S1.

It is generally believed that TAS2Rs possess only one binding site for both agonists and antagonists to bind (Behrens and Meyerhof, 2013), and it’s the type of interactions with selected residues in the binding site that determines whether the ligand is of agonistic or antagonistic nature (Liu et al., 2018). However, it has been recently suggested that, similar to class A GPCRs, TAS2Rs also possess an additional vestibular binding site that are transiently occupied by agonists. For example, TAS2R46, which has a broad agonist diversity, turned out to feature not only the orthosteric binding site, but also a second vestibular site, located above the orthosteric site. This two-site architecture might play a role as an access control to discriminate the highly structurally diverse agonists of TAS2R46 (Sandal et al., 2015). In addition, TAS2Rs can also achieve multi-specification toward a vast range of chemical structures by forming different types of interaction with different ligands (Figure 1) (Brockhoff et al., 2010; Sakurai et al., 2010; Born et al., 2013; Marchiori et al., 2013; Di Pizio et al., 2018; Nowak et al., 2018; Schneider et al., 2018).

The interaction between TAS2Rs and bitter tastants at the binding site is dictated by multiple factors including the type of ligands, the membrane lipids and the movements of TMs and ECLs (Liu et al., 2018). Among these factors, the cholesterol components in the cell membrane are particularly important, because the majority of human TAS2Rs has been found to consist of a cholesterol interaction amino acid motif (LxxYxxK/R) that affects the location and function of TAS2Rs in the cell membrane where cholesterols aggregate to form caveolae (Jafurulla et al., 2011). It has also been shown that mice deficient of caveolin-1 (a protein for forming caveolae) exhibit markedly impaired response to bitter substance (e.g., chloroquine) in terms of relaxing the pre-contracted aorta (Manson et al., 2014), suggesting that membrane lipid and caveolae are the essential players in the TAS2R-mediated signal transduction.

## Distribution and Function of TAS2Rs in Cardiac and Smooth Muscle Cells

**FIGURE 2:**
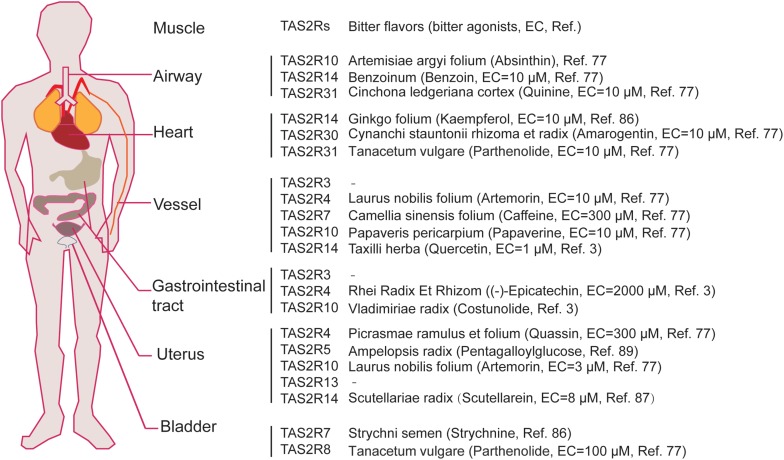
The expression map of TAS2Rs in different muscle tissues which can be activated by bitter components from bitter flavored TCM. EC represents efficiency concentration.

Over the past decade, studies have shown that TAS2Rs are expressed in diverse types of muscle cells including cardiac muscle cells (cardiomyocytes), and SMCs in various organs such as blood vessels, pulmonary airways, gastrointestinal tracts, and so on (Table 3; Foster et al., 2014b; Shaik et al., 2016; Lu et al., 2017). It is clear that the distribution and expression of TAS2Rs in different kinds of muscle cells vary considerably. However, there appears a certain pattern in all kinds of muscle cells that TAS2R3, 4, 5, 10, 13, 19, and 50 are always expressed at moderate levels and TAS2R14 is always expressed at high level (Jaggupilli et al., 2017). Intriguingly, studies have shown that TAS2Rs can mediate either relaxation of SMCs (e.g., in airway, bladder, and uterus) (Liggett, 2014; Pan et al., 2017; Kim et al., 2018), or contraction of SMCs [e.g., in pulmonary artery (Upadhyaya et al., 2014) and gastrointestinal tract (Avau et al., 2015)]. Interestingly, the bitter components from TCM are often known to activate TAS2Rs expressed in different kinds of muscle cells (Figure 2). Therefore, it is highly possible to explore the vast source of bitter flavored TCM for extra-oral TAS2R agonists as specific muscle relaxation agents based on their expression profiles in different muscle tissues.

**TABLE 3:** The distribution of TAS2Rs in different muscle tissues.

**Species**	**Cardial muscle**	**Airway smooth muscle**	**Pulmonary artery smooth muscle**	**Vascular smooth muscle**	**Gastrointestinal smooth muscle**	**Uterine smooth muscle**	**Bladder smooth muscle**
Human	TAS2R14, TAS2R31, TAS2R30, TAS2R19, TAS2R13, Foster et al.,2013	TAS2R10, TAS2R14, TAS2R31, Deshpande et al.,2010	TAS2R3, TAS2R4, TAS2R14, TAS2R10, Manson et al., 2014^*^	TAS2R3, TAS2R4, TAS2R7, TAS2R10, TAS2R14, TAS2R39, TAS2R40, Chen et al., 2017^*^	TAS2R3, TAS2R4, TAS2R10, Avau et al., 2015^*^	TAS2R14, TAS2R5, TAS2R10, TAS2R4, TAS2R13, Zheng et al.,2017	TAS2R7, TAS2R8, TAS2R13, TAS2R1, TAS2R9, Zhai et al.,2016
Rat	rTAS2R143, rTAS2R126, rTAS2R135, rTAS2R121, rTAS2R120, Foster et al.,2013			rTAS2R39, rTAS2R40, rTAS2R108, rTAS2R130, rTAS2R137, rTAS2R140, Chen et al., 2017^*^			
Mouse					mTAS2R108, mTAS2R135, mTAS2R137, Avau et al., 2015^*^	mTAS2R135, mTAS2R143, mTAS2R126, Zheng et al.,2017	mTAS2R144, mTAS2R138, mTAS2R117, mTAS2R130, mTAS2R114, Zhai et al.,2016

*The rank order of TAS2Rs are according to their expression level except for labeled with asterisk.*

### TAS2Rs in Cardiac Muscle Cells

Contraction of cardiac muscle cells (cardiomyocytes) determines the pump function of the heart, but too fast contraction leads to ventricular tachycardia. In mice it has been shown that TAS2Rs agonists such as denatonium and quinine can inhibit cardiac contraction induced by electrical field/epinephrine-stimulation via TAS2Rs activation (Foster et al., 2014a). The expression map of TAS2Rs in cardiomyocytes from humans, rats and mice are significantly different. In neonatal rat cardiomyocytes, it has been shown that the top five genes for TAS2Rs are *TAS2R143, 126, 135, 121, 120* (in the order of mRNA expression level) (Foster et al., 2013). In adult rat cardiomyocytes, however, the top three genes are *TAS2R120, 143, 121* (in the order of mRNA expression level) (Xin et al., 2018). In human cardiomyocytes, *TAS2R14, 31, 30, 19, 13* are the top five TAS2Rs (in the order of mRNA expression level) (Foster et al., 2013). Additionally, the expression levels of TAS2Rs in cardiomyocytes are dynamic during heart development. For example, in rats *TAS2R120, 121* increased their expressions nearly 20-fold during development and changed from being the last two of the top five genes of TAS2Rs in neonatal cardiomyocytes to become the dominating first and third top ones in adult cardiomyocytes (Foster et al., 2013). Therefore, TAS2Rs agonists may have important inotropic effects on cardiomyocytes, which can be useful in development of pharmacological tools for the treatment of ventricular tachycardia.

### TAS2Rs in Airway Smooth Muscle Cells

Airway smooth muscle cells control the diameter of the pulmonary airways by contraction/relaxation. Hypercontraction of ASMCs will lead to airway constriction and obstruction which is the cardinal character of asthma. Bronchodilators are central in the treatment of airway hypercontractile diseases such as asthma and chronic obstructive pulmonary disease (COPD). In human ASMCs, it has been shown that TAS2R10, 14, 31 are the three most highly expressed subtypes of TAS2Rs, and activation of the TAS2Rs by bitter tastants induces significant bronchodilatory effect (Deshpande et al., 2010). In addition, the subtype of TAS2R5 is also considered to have a prime role in bronchodilation just like TAS2R10, 14, although it is expressed at a much lower level (Grassin-Delyle et al., 2013). Together, bitter agonists are considered as a novel class of bronchodilators in treatment of obstructive airway diseases such as asthma and COPD (Orsmarkpietras et al., 2013).

### TAS2Rs in Vascular Smooth Muscle Cells

Vascular smooth muscle cells (VSMCs) regulate the caliber of blood vessels and associated blood pressure by contraction/relaxation. It is reported that TAS2Rs are expressed in VSMCs of aorta, pulmonary artery, and system artery, and bitter agonists can exert profound vascular activities including dilation and antagonism of α-adrenoceptors, as described below.

In guinea pigs, bitter agonists for TAS2R3, 4, 10, and 14 have been shown to induce strong relaxation in phenylephrine pre-contracted aorta (Manson et al., 2014). In human VSMCs the expression of TAS2R46 is confirmed, and in rats the intravenous injection of denatonium (TAS2R agonist) leads to a transient drop in blood pressure (Lund et al., 2013). Additionally, the activation of TAS2Rs by either denatonium or quinine (also a known TAS2R agonist) can reduce the tension of pre-contracted rat aorta (Xin and Chen, 2017).

In human pulmonary artery, Upadhyaya et al. (2014) reported that 21 subtypes of TAS2Rs are expressed. Interestingly, this study showed that bitter agonist, dextromethorphan induces vasoconstriction via a TAS2R1-mediated Ca^2+^ response in human pulmonary arterial VSMCs. Other bitter agonists including chloroquine (ChQ) and noscapine, however, are shown to mediate relaxation of human pulmonary arteries (Manson et al., 2014).

In rat mesenteric and cerebral arterial VSMCs, *TAS2R108, 130, 137, 139, 140* are shown to be expressed while in human omental arterial VSMCs, *TAS2R3, 4, 7, 10, 14, 39, 40* are expressed, and activation of these TAS2Rs by CHQ and quinine relaxes rat mesenteric and cerebral arteries and human omental arteries (Chen et al., 2017). Therefore, TAS2R agonists may be useful pharmacological tools for treatment of hypertension.

### TAS2Rs in Gastrointestinal Smooth Muscle Cells

Gastrointestinal smooth muscle cells (GSMCs) via their contraction and relaxation are essential for either maintaining the normal motility of the gastrointestinal tract during the process of digestion (Sanders et al., 2012), or causing the hyper/hypo motility of the gastrointestinal tract that can lead to diarrhea/constipation (Drossman, 2016). Although in GVSMCs TAS2Rs are shown to be expressed, but their function remains controversial (Avau and Depoortere, 2016). In human GSMCs, it was reported that *TAS2R3, 4, 10* are expressed, and in mouse GSMCs *TAS2R108, 135, 137* are expressed (Avau et al., 2015). However, it appears that TAS2R agonists such as denatonium can induce either contraction or relaxation of GSMCs, depending on not only the agonist concentration but also the region of the gastrointestinal tract (Avau et al., 2015). It has also been shown in mice that the gastric muscle relaxation induced by TAS2R agonists such as denatonium and phenyltiocarbamide (PTC) can be correlated with the decreased hunger and increased satiety ratings after a meal, which shows potential of targeting TAS2Rs for decreasing caloric intake (Avau et al., 2015; Deloose et al., 2017).

These observed TAS2R-mediated contractility or relaxation of GSMCs suggest possibilities of treating gastrointestinal motility diseases such as ileus and constipation with bitter agonists. In addition, the effect of bitter tastants on gastric emptying and hence on satiation may encompass a therapeutic potential in the treatment of obesity.

### TAS2Rs in Uterine Smooth Muscle Cells

Uterine smooth muscle cells (USMCs) relax and contract to a great extent during pregnancy and child birth, but that there are few measures to prevent and treat unwanted contraction of USMCs remains a central feature of preterm birth (PTB) which is the leading cause of neonatal mortality and morbidity. Therefore, identifying novel targets for tocolytics are essential for more successful management of PTB. In a recent study, it has been shown that in human and mouse USMCs, TAS2R4, 5, 10, 13, 14 and their canonical signaling components (gustducin, PLCβ) are expressed (Zheng et al., 2017). Furthermore, bitter compound, ChQ at 10 mM can induce [Ca^2+^]_i_ rise and completely relax human USMCs pre-contracted by different uterotonics, which is mediated though TAS2R14 but not TAS2R10. Therefore, targeting TAS2Rs may be an attractive approach to developing effective tocolytics for PTB management.

### TAS2Rs in Bladder Smooth Muscle Cells

Bladder smooth muscle cells (BSMCs) contract and relax to control the disposal of urine and the overactive dysfunction of them will lead to bladder syndrome characterized by the presence of incontinence, frequency, and nocturia, which has serious effects on quality of life. Recently, it is reported that TAS2R7, 8 are the most abundant TAS2Rs in human BSMCs. Additionally, the activation of TAS2Rs inhibited spontaneous and electrical field stimulation-induced contraction of BSMCs and relaxed carbachol- and KCl-induced contractions of BSMCs (Zhai et al., 2016). Thus, TAS2Rs may be a new target to develop drugs for overactive bladder symptoms.

Taken together, the functional implications of TAS2Rs widely dispersed in various types of muscle cells shed light on discovering of muscle relaxant from bitter flavored TCM (Gilca and Barbulescu, 2015).

## Signaling Mechanisms of TAS2Rs in Different Muscle Cells

**FIGURE 3:**
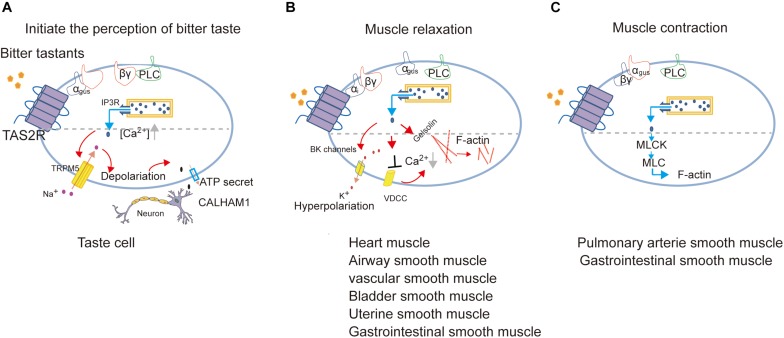
Proposed model of signal transduction of TAS2Rs in different cell types to mediate the perception of bitter taste (adapted from Behrens and Meyerhof, 2015; Avau and Depoortere, 2016; Shaik et al., 2016; Lu et al., 2017) **(A)**, muscle relaxation (adapted from Zhai et al., 2016; Kim et al., 2017; Pan et al., 2017; Zheng et al., 2017; An and Liggett, 2018; Scadding, 2018) **(B)** or muscle contraction (adapted from Upadhyaya et al., 2014; Avau et al., 2015) **(C)**.

It is important to note that bitter tastants can induce signaling that diverges to execute different biological roles depending on cell types (Figure 3). There are three different signaling cascades following Ca^2+^ signaling which induce the perception of bitter taste, muscle relaxation, and muscle contraction, respectively.

In cardiomyocytes, TAS2Rs agonists inhibit cardiac contractions by attenuating the voltage-dependent calcium channels (VDCC), and the consequent Ca^2+^ release by ryanodine receptors (Mak and Hanania, 2012; Foster et al., 2014a; Xin et al., 2018).

In ASMCs, Kim et al. identified the role of G_αi_ in transmitting TAS2R signaling and found a very low expression of G_αgust_, which together indicates that TAS2Rs can couple to different G_α_ in a cell-type dependent manner (Kim et al., 2017; An and Liggett, 2018). Furthermore, Deshpande et al. (2010) proposed that the activation of TAS2Rs induces a microdomain [Ca^2+^]_i_ response close to the cell membrane, which opens large-conductance Ca^2+^-activated K^+^ (BK) channels, leading membrane hyperpolarization and muscle relaxation (Deshpande et al., 2010). But further direct measurement of BK channel currents indicates that bitter tastants induce relaxation of ASMCs not through BK channels (Zhang et al., 2012) but instead by inhibiting L-type VDCC to decrease [Ca^2+^]_i_ (Deshpande et al., 2010; Zhang et al., 2013; Xin et al., 2018).

Gelsolin is a calcium-activated actin-severing and -capping protein found in ASMCs and plays a critical role in ASMC relaxation. Mikami et al. (2017) found that the activation of gelsolin may contribute to relaxation induced by bitter tastants. Another experiment reported that bitter agonists (denatonium and PTC) attenuated acetylcholine-induced contraction via inhibiting the phosphorylation of myosin light chain (MLC; Sakai et al., 2016).

In human pulmonary arterial VSMCs, Upadhyaya et al. (2014) proposed that the calcium increase from the canonical TAS2Rs signaling pathway directly activates MLC kinase and subsequently increases the phosphorylated MLC, leading to constriction of pulmonary artery.

In USMCs (Zheng et al., 2017) and GSMCs (Avau et al., 2015), the candidate TAS2R-coupled G-protein such as G_αgust_, PLC and TRPM5 are also expressed which indicates that the canonical TAS2R signaling pathway may function in regulating the contraction/relaxation of SMCs in uterus and gastrointestinal tract.

## Molecular Receptive Ranges of TAS2Rs

To date, based on *TAS2Rs* heterologous expression system in HEK293 or insect Sf9 cell line, 21 of the 25 *TAS2Rs* except for *TAS2R19, 42, 45, 60* (Bufe et al., 2002; Behrens et al., 2004; Kuhn et al., 2004; Brockhoff et al., 2007; Meyerhof et al., 2010; Roland et al., 2011) and 21 of the 35 *Tas2rs* (hereafter, gene symbol: *TAS2R* for humans, *Tas2r* for rats and mice) have been found to have specific agonists (Lossow et al., 2016). Many TAS2Rs have a wide range of recognition of bitter tastants, leading humans to respond to 1000 of diverse bitter compounds (Meyerhof et al., 2010; Lossow et al., 2016). The top 6 of 25 *TAS2Rs* according to receptive profile are *TAS2R14* > *TAS2R10* > *TAS2R1* > *TAS2R46* > *TAS2R4* > *TAS2R38*, and all these *TAS2Rs* have homologous genes in rats and mice (Suku et al., 2017). It was reported that three *TAS2Rs* (*TAS2R10, 14, 46*) can detect 50% of all the tested bitter compounds (Sainz et al., 2007; Brockhoff et al., 2011; Levit et al., 2014; Jaggupilli et al., 2016; Nowak et al., 2018). The top 4 of 35 mouse *Tas2rs* (*mTas2rs*) according to receptive range are *mTas2r10*5 > *mTas2r144* > *mTas2r121* > *mTas2r135*. Among them, *mTas2r121, 135, 144* have specific homologous genes in human with *TAS2R13, 60, 40*, respectively (Lossow et al., 2016). Therefore, a few of TAS2Rs play a more important role in mediating the function of bitter compounds, considering the relatively high expression in different muscle tissues (Table 3) and wide receptive ranges (Supplementary Table S1).

TAS2Rs with pronounced amino acid sequence differences can have agonists in common even though they recognize similar compounds by different binding modes (Born et al., 2013; Dagan-Wiener et al., 2019). An extensive study on 97 bitter compounds comprising flavonoids and isoflavonoids have identified 68 activating TAS2R14, and 70 activating TAS2R39 but 58 overlapping to activate both TAS2R14 and TAS2R39, although they are not closely related (Roland et al., 2011, 2013; Lossow et al., 2016). Evidence that TAS2Rs overlap in ligand specificity also suggests that they may cause joint effects (Di Pizio and Niv, 2015). Recent findings show that orthologous TAS2Rs in humans, rats and mice do not share conserved agonists (Lossow et al., 2016).

Studies have shown that structurally diverse bitter components from bitter flavored TCM also can activate TAS2Rs. The general profiles of TAS2Rs agonists from bitter flavored TCM were summarized in Supplementary Table S1 according to ETCM: an encyclopedia of TCM (Tang et al., 2018) and BitterDB (Born et al., 2013; Dagan-Wiener et al., 2019). It is shown that 16 of 25 TAS2Rs have natural agonists from bitter flavored TCM. Among them, TAS2R10, 14, 39, 46 can be activated by more than 20 bitter components from bitter flavored TCM, indicating that these TAS2Rs may be the primary functional targets of bitter flavors (Figure 1A). Figure 1B shows the profile of bitter tastants from bitter flavored TCM which can activate a number (from 1 to 9) of TAS2Rs. It is clear that the majority of the bitter components activate either one (30.16%) or two (39.68%) TAS2Rs, and only a small portion of them can activate more than two TAS2Rs. For example, picrotoxinin derived from *Artemisiae argyi* folium can activate 5 TAS2Rs (TAS2R1, 10, 14, 46, 47), which indicates that *A. argyi* folium may have a wide range of functions in different tissues (Behrens et al., 2004). Incidentally, ∼50% of the bitter components from bitter flavored TCM with specific TAS2Rs are derived from Asteraceae (Figure 1C).

## Bitter Components Extracted From Bitter Flavored TCM

Many bitter components extracted from bitter flavored TCM such as salicin and quinine have been identified with their specific TAS2Rs (Supplementary Table S1) and developed for treating diverse diseases (Soares et al., 2013; Scadding, 2018).

Intriguing, among the 625 materials of TCM reported in *the 2015 edition of the Chinese Pharmacopoeia*, 241 of them are bitter flavors and are mostly derived from plants (Pharmacopoeia Committee of the People’s Republic of China, 2015). According to their action dogma, the function of some TCM prescriptions such as hemostatic, blood-activating, stasis-dispelling medicinal, cough-suppressing, panting-calming medicinal, purgative, digestant medicinal, and tocolytics, labor promoting medicinal may be correlated with regulation of the muscle function in cardiovascular system, lung, gastrointestinal tract, and uterus, respectively (Leem et al., 2018). In this review, we show the bitter flavored TCM and their main components with related properties, which are manually selected according to the following criteria: (a) with the above mentioned action recorded in *The Pharmacopoeia of the People’s Republic of China* (PPRC) 2015 *Edition* (Pharmacopoeia Committee of the People’s Republic of China, 2015) and the *National Compilation of Chinese Herbal Medicine (NCCHM)* (Wang, 2014). (b) the components of bitter flavors and some correlated TAS2Rs are clarified in the database of ETCM (Tang et al., 2018) and BitterDB (Dagan-Wiener et al., 2019), respectively (Supplementary Tables S2–S5).

Specifically, Supplementary Table S2 shows 32 bitter flavored TCM with hemostatic, blood-activating, and stasis-dispelling functions, which are correlated with contraction/relaxation of cardiovascular SMCs. Structurally diverse range of bitter components have been identified from these 32 TCMs. Among them, naringenin (TAS2R14), quercetin (TAS2R14), scutellarin (TAS2R14, 39), (-)-epicatechin (EC) (TAS2R4, 5, 39), and kaempferol (TAS2R14, 39) can stimulate specific TAS2Rs (Dagan-Wiener et al., 2019). Since TAS2R4 and TAS2R14 are the abundantly expressed TAS2Rs in cardiovascular systems, these bitter components may function to relax cardiovascular SMCs via activating TAS2R signaling (Foster et al., 2013).

Supplementary Table S3 shows the bitter flavored TCM with function to calm panting and suppress coughing, which have been reported to regulate the contractility of ASMCs. α-thujone (TAS2R10, 14), apigenin (TAS2R14, 39), absinthin (TAS2R10, 14, 46, 47), benzoin (TAS2R10, 14), camphor (TAS2R4, 10, 14, 47), dihydroxychalcone (TAS2R14, 39), epicatechin (TAS2R4, 5, 39), (-)-epicatechin (EC) (TAS2R4, 5, 39), flavone (TAS2R14, 39), kaempferol (TAS2R14, 39), quercetin (TAS2R14), and taurocholic acid (TAS2R4) extracted from these bitter flavored TCM have been found to activate one of the functional TAS2Rs (TAS2R5, 10, and 14) which may mediate the relaxation of ASMCs (Deshpande et al., 2010; Dagan-Wiener et al., 2019).

Interestingly, a recent study performed on a database of medicinal plants established a positive association between bitter herbs and “asthma relief” activity (Gilca and Barbulescu, 2015; Dragos and Gilca, 2018). We also found that naringin extracted from *Citrus paradisi* (Wang et al., 2016) and artesunate extracted from *Artemisia annua* (Wang et al., 2018) reduced airway resistance in ovalbumin (OVA)-treated mice *in vivo*, and reduce traction force of ASMCs *in vitro* most likely via TAS2Rs. These findings provide important evidence that naringin and artesunate may be bronchodilators for treating asthma. Yu et al. (2017) investigated the protective mechanisms of bitter total flavonoids from *Selaginella uncinata* on airway hyperresponsiveness in a rat model of OVA-treated asthma. They demonstrated that total flavonoids exerted anti-inflammatory function through the activation of TAS2R10.

Supplementary Table S4 shows the bitter flavored TCM with purgative and digestant functions which are correlated to regulate the contractility of GSMCs and thus the peristalsis of gastrointestinal tract. Intriguing is that some of the bitter flavored TCM have opposite effect on relaxation/contraction of gastrointestinal tract depending on the concentration, consistent with the reported effect of TAS2R signaling in GSMCs (Avau et al., 2015). Many of the components extracted from these bitter flavored TCM such as costunolide (TAS2R10, 14, 46), (-)-epicatechin (EC) (TAS2R4, 5, 39), flavone (TAS2R14, 39), kaempferol (TAS2R14, 39), luteolin (TAS2R14, 39), naringenin (TAS2R14), papaverine (TAS2R7, 10, 14), quassin (TAS2R4, 10, 14, 46), quercetin (TAS2R14), and scutellarein (TAS2R14, 39) can activate some of the functional TAS2Rs in GSMCs (Dagan-Wiener et al., 2019). Since TAS2R4, 10 are the abundantly expressed bitter receptors in these cells, these bitter components may function on targeting bitter receptors to regulate the contractility of GSMCs (Avau et al., 2015).

Versatile plants exhibit biological activity that targets against uterine muscle contractility (Gruber and O’Brien, 2011). Atractylodis macrocephalae, Inulae radix, Scutellariae radix, Taxilli herba, and Visci herba are the five bitter flavored TCM which have anti-abortion effect, but Leonurus herba and Verbenae herba have the uterotonic effect recorded in PPRC/NCCHM (Supplementary Table S5). Interesting is that quercetin extracted from Taxilli herba, scutellarein extracted from Scutellariae radix and homoeriodictyol extracted from Visci herba can activate TAS2R14 (Dagan-Wiener et al., 2019). Considering TAS2R14 is the abundantly expressed TAS2R in USMCs (Zheng et al., 2017), these bitter components may suppress (tocolytic agents) or induce (uterotonic agents) uterine contractions.

Magnesium sulfate has long been used for fetal neuroprotection (Jayaram et al., 2018) and to delay preterm labor (Escobar et al., 2019) in clinical practice with unclear molecular targets. Interestingly, magnesium sulfate is called bitter salts in TCM. Very recently, it is shown that TAS2R7 is the only receptor for bitter salt such as magnesium sulfate and manganese chloride (Behrens et al., 2019), perhaps correctly inferring that magnesium sulfate may function as a tocolytic agent via TAS2R7 signaling.

## Bioinformatics-Aided Screening of Bitter Flavored TCM

In the last decade, many structurally diverse bitter tastants have been found to evoke the signaling of TAS2Rs in several types of muscle cells which are correlated with diverse physiological and pathological events (Fletcher et al., 2017), implying that bitter agonists may be novel potential drug agents. Additionally, Di Pizio et al. (2019) used Lipinski’s Rule of 5 to analyze the bitter compounds from BitterDB, and found that the majority of the bitter compounds can be considered drug-like. So far, however, there are no drugs approved yet, based on targeting TAS2Rs. Part of the reason can be attributed to the low affinity of bitter tastants with TAS2Rs. Thus, they often work at mid-to-high micromolar concentration, and such potency against TAS2Rs is practically insufficient for repurposing them to treat TAS2R-correlated decreases. Therefore, it is desirable to explore the widely available bitter tastants from bitter flavored TCM toward identification of more potent TAS2R agonist as drug agents for muscle relaxation therapy (Di Pizio et al., 2019; Lee et al., 2019).

**FIGURE 4:**
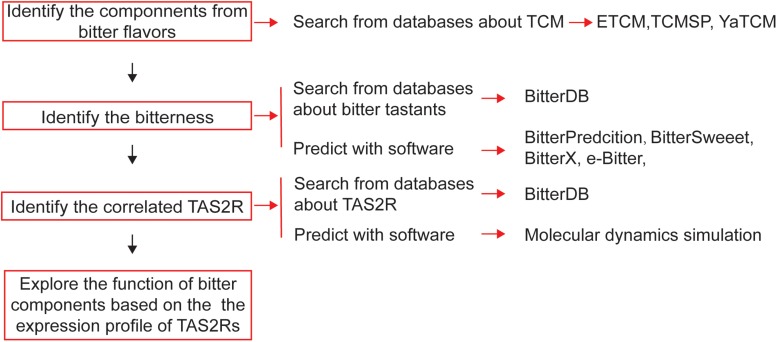
Schematic diagram of the process of bioinformatics-aided *in silico* analysis of bitter flavored TCM from databanks in order to develop novel drugs for regulating muscle relaxation/contractility.

For screening bitter components from the vast source of bitter flavored TCM, bioinformatics is a very useful tool to be employed (Figure 4). The first step for drug discovery based on bitter flavored TCM is to understand their function and to identify the main components. Recently, many online databases about TCM and their components have been established [e.g., ETCM (Tang et al., 2018), TCMSP (Ru et al., 2014), and YaTCM (Li et al., 2018)], which are easy to use to clarify the type, function, and components of TCM (Table 4).

**TABLE 4:** Databases and software for modern drug discovery based on bitter flavored TCM.

**Database/Software**		**Description**	**Web address**	**References**
TCM and their components	Acupuncture.com.au	Classification of TCM formulations based on their actions	http://www.acupuncture.com.au/	Lukman et al.,2007
	Dictionary of Chinese Herbs	Disease-specific TCM formulations, toxicity and side effects	http://alternativehealing.org/	Lukman et al.,2007
	Plants For a Future	TCM herbs with their potential side effects, physical characteristics, and medicinal usages substantiated by relevant scientific citations	http://www.pfaf.org	Lukman et al.,2007
	TCM Knowledge Base Grid	TCM medicine database, traditional Chinese drug database, TCM literature databases, traditional Tibetan drug database	http://www.cintcm.com	Lukman et al.,2007
	ETCM	ETCM includes comprehensive and standardized information for the commonly used herbs and formulas of TCM, as well as their ingredients. The herb basic property and quality control standard, formula composition, ingredient drug-likeness, as well as many other information provided by ETCM can serve as a convenient resource for users to obtain thorough information about a herb or a formula	http://www.nrc.ac.cn:9090/ETCM/	Tang et al.,2018
	BATMAN-TCM	BATMAN-TCM main functions include: (1) TCM ingredients’ target prediction, (2) functional analyses of targets including biological pathway, Gene Ontology functional term and disease enrichment analyses, (3) the visualization of ingredient-target-pathway/disease association network and KEGG biological pathway with highlighted targets, and (4) comparison analysis of multiple TCMs	http://bionet.ncpsb.org/batman-tcm/	Behrens et al.,2019
	YaTCM	YaTCM is a free web-based toolkit, which provides comprehensive TCM information and is furnished with analysis tools	http://cadd.pharmacy.nankai.edu.cn/yatcm/home	Li et al.,2018
	TCMSP	TCMSP was built based on the framework of systems pharmacology for herbal medicines	http://lsp.nwu.edu.cn/browse.php?qc=herbs	Ru et al.,2014
Bitter tastants	BitterDB	BitterDB now holds over 1000 bitter molecules and provides a unique platform for structure-based studies with high-quality homology models and known ligands	http://bitterdb.agri.huji.ac.il	Dagan-Wiener et al.,2019
Bitterness prediction	BitterPredict	BitterPredict predicts whether a compound is bitter or not, based on its chemical structure, which is a machine learning classifier	http://bitterdb.agri.huji.ac.il	Dagan-Wiener et al.,2017
	BitterX	BitterX is a web server on bitter compound identification and potential target prediction for small molecule compounds	http://mdl.shsmu.edu.cn/BitterX/	Huang et al.,2016
	e-bitter	Predict the bitterness with machine learning models		Tuwani et al.,2018
	BitterSweet Forest	Predict the dichotomy of bitter–sweet taste		Jayaram et al.,2018
TAS2R prediction	GOMoDo	GOMoDo is a web server to seamlessly model GPCR structures and dock ligands to the models in a single consistent pipeline	http://molsim.sci.univr.it/gomodo	Sandal et al.,2013

While 100 and 1000 of components have been clarified from bitter flavored TCM, their bitterness needs to be determined because we still do not know the bitter coding of bitter tastants and thus cannot intuitively take the component derived from bitter flavored TCM as being bitter itself. Since experimental-screening of the bitter tastants is very expensive and laborious, bitter prediction methods *in silico* receive much attention recently. There have been some software developed to predict the bitterness of one chemical compound (Table 4), achieved by using either ligand based, structure based or machine learning based methods (Bahia et al., 2018). For example, BitterPredict is a software for predicting the bitterness of compounds based on machine learning, which predicted that about 40% of random molecules, a large portion (66%) of clinical and experimental drugs, and natural products (77%) are bitter (Dagan-Wiener et al., 2017). BitterX (Huang et al., 2016) and e-Bitter (Zheng et al., 2018) are two open-access software for bitter prediction also based on machine learning. Interestingly, BitterSweet Forest (Tuwani et al., 2018) can predict the dichotomy of bitter–sweet taste. Results obtained by using these tools so far are encouraging, which may promote wider use of such reliable tools for bitter prediction.

Then, we also need to determine the receptors for these newly clarified bitter components. Similar to the prediction of bitterness, computational methods can be used to predict the TAS2Rs of bitter components, even the crystal structure of TAS2Rs and the recognition mechanisms are still unknown (Katritch et al., 2013). Techniques such as homology modeling, molecular docking (Sousa et al., 2013), and molecular dynamics simulation could in principle provide insights into the 3D structure of TAS2Rs and agonist/antagonist binding (Roland et al., 2013; Suku et al., 2017). Using homology modeling, molecular docking, and point mutagenesis experiments, Nowak et al. (2018) investigated the architecture of the TAS2R14 binding pocket and found that TAS2R14 provides a large number of agonist-selective contact points likely exceeding that of all other promiscuous TAS2Rs.

It is worthy to note that, although some web tools such as GOMoDo (Sandal et al., 2013) have been developed to seamlessly model GPCR structures and dock ligands to the models, homology model and molecular docking are still not suitable for TAS2R prediction due to the low sequence identity shared by TAS2Rs with the available GPCR templates and only low resolution homology models accessible (Beuming and Sherman, 2012). In addition, most docking algorithms neglect the presence of explicit solvent, even though water molecules may be crucial to stabilize the ligand in a variety of membrane proteins. In order to overcome this issue, methods that increase the sampling of the conformational space, such as flexible docking (Brockhoff et al., 2010; DiPizio and Niv, 2014; Karaman et al., 2016; Nowak et al., 2018; Thawabteh et al., 2019) or molecular dynamics (Sandal et al., 2015; Chen et al., 2018; Liu et al., 2018; Jaggupilli et al., 2019) can be used. Recently, the predictions are improved by molecular dynamics simulation approaches from all atom and coarse grained to hybrid methods bridging the two scales, which have provided exciting functional insights into TAS2Rs (Schneider et al., 2018). For example, subnanosecond all atom/molecular dynamics (AA/MD) simulation has been applied to study antibiotic binding to TAS2R7 (Liu et al., 2018), as well as TAS2R4, 14, and 20 (Jaggupilli et al., 2019). Beside AA/MD, hybrid molecular mechanics/coarse grained (MM/CG) simulations (Leguebe et al., 2012; Marchiori et al., 2013; Sandal et al., 2015; Schneider et al., 2018), used for soluble and membrane proteins, have been tailored for low resolution GPCR models, such as TAS2Rs. This approach has been applied to three ligand/TAS2R complexes so far, clearly improving the quality of the predictions (Fierro et al., 2017).

When combining these computational methods, the chemical property of components from TCM and the potential bitter receptors can be predicted which will promote the screening functional bitter compounds from large amount of bitter flavored TCMs. Nevertheless, these results predicted by informatics require further confirmation in experimental studies.

## Discussion and Future Directions

Bitter components extracted from TCM cannot only be used for the treatment of disease but also a great resource for developing new modern drugs. To screen muscle relaxants based on the distribution and function of TAS2Rs in muscle tissues, it may be more efficient to directly screen from these well-known bitter flavored TCM. On the other hand, we need to pay attention to the side effects of bitter tastants for drugs since many bitter tastants are toxins. In fact, the extra-oral expression of TAS2Rs has been hypothesized to cause off-target effects of bitter medications (Clark et al., 2012; Gilca and Dragos, 2017). Therefore, the suitable dose will be critical for developing drugs based on bitter tastants.

We also need to carefully assess the therapy effect of bitter tastants for different individuals, considering there may be big differences in genetic expression types and abundance of TAS2Rs from person to person (Yoon et al., 2016). It is also well known that children are more sensitive to bitter tastants than adults (Mennella et al., 2014). These indicate that each individual may respond to bitter substance differently, which may influence the medical function of these bitter tastants.

Additionally, TAS2Rs polymorphisms are a common phenomenon in different species. For example, both the gene types and recognizing profiles are significantly different regarding the same tissue in humans, rats and mice. So that, the bitter compounds, β-glucopyranosides and PTC that elicit strong bitter taste in humans, are tasteless at all to mice (Meyerhof, 2005). This means that the function of bitter tastants acquired from animal studies cannot be directly referenced for functioning in humans.

Finally, in the application of TCM, it is found that usually the mixture of various substances produces more potent effect compared to a single component. However, promiscuity is common in bitter compound so that some bitter compounds can be agonistic to one TAS2R but antagonistic to another TAS2Rs. Therefore, it is unknown whether bitter-compound mixtures exert suppression and/or synergistic effects (Brockhoff et al., 2011; Roland et al., 2014), until they are thoroughly clarified, which is absolutely need in the development of bitter compound-based drugs.

## Author Contributions

ML and LD conceived and designed the study. ML wrote the manuscript. KN, YJ, and ZY collected some data for this manuscript. LD revised the manuscript.

## Conflict of Interest Statement

The authors declare that the research was conducted in the absence of any commercial or financial relationships that could be construed as a potential conflict of interest.
