# Supplementary material for: Toward the Identification of Extra-Oral TAS2R Agonists as Drug Agents for Muscle Relaxation Therapies via Bioinformatics-Aided Screening of Bitter Compounds in Traditional Chinese Medicine
Source: Front Physiol. 2019 Jul 16;10:861. doi: 10.3389/fphys.2019.00861 (PMC6647893; doi:10.3389/fphys.2019.00861)
Supplement: Supplementary file 1 [file Data_Sheet_1.docx]

Supplementary data

Towards the Identification of Extra-oral TAS2R Agonists as Drug Agents for Muscle Relaxation Therapies via Bioinformatics-aided Screening of Bitter Compounds in Traditional Chinese Medicine

LUO Mingzhi ^1^, NI Kai ^1^, JIN Yang ^2^, YU Zifan ^1^, DENG Linhong ^1,*^

*1. Changzhou Key Laboratory of Respiratory Medical Engineering, Institute of Biomedical Engineering and Health Sciences, Changzhou University, 1 Gehu Road, Wujin District, Changzhou 213164, China*

*2. Bioengineering College, Chongqing University, Chongqing* *400044, China*

**Table S1 The activation map of TAS2Rs with different bitter components from TCM ([**[**1**](#_ENREF_1)**;** [**2**](#_ENREF_2)**; 3])**

| No. | TAS2R | Agonist | TCM |
| --- | --- | --- | --- |
| 1 | TAS2R1 | Amarogentin | Gentiana manshurica, Swertiae herba, Cynanchi Stauntonii rhizoma et radix |
|  |  | Arborescin | Artemisia myriantha |
|  |  | Cascarillin | Rhamnus purshiana cortex |
|  |  | Humulone isomers | Humulus lupulus |
|  |  | Parthenolide | Tanacetum vulgare |
|  |  | Picrotoxinin | Anamirta cocculus fructus,  Artemisiae argyi folium |
| 2 | TAS2R3 | - |  |
| 3 | TAS2R4 | Arborescin | Artemisia myriantha |
|  |  |  |  |
|  |  | Artemorin | Laurus nobilis folium |
|  |  | Brucine | Strychni semen |
|  |  | Camphor | Cinnamomum Camphora rhizoma |
|  |  | Colchicine | Lilii bulbus |
|  |  | (-)-Epicatechin (EC) | Ginkgo semen, Rhei radix et rhizoma |
|  |  | Parthenolide | Tanacetum vulgare |
|  |  | Quassin | Picrasmae ramulus et folium |
|  |  | Quinine | Cinchona ledgeriana cortex |
|  |  |  |  |
| 4 | TAS2R5 | (-)-Epicatechin (EC) | Ginkgo semen, Rhei radix et rhizoma |
|  |  | Pentagalloylglucose (PGG) | Ampelopsis radix, Paeoniae radix alba, Canavaliae semen, Terminaliae fructus, Moutan cortex |
|  |  |  |  |
| 5 | TAS2R7 | Caffeine | Camellia sinensis folium |
|  |  | Papaverine | Papaveris pericarpium |
|  |  | Quinine | Cinchona ledgeriana cortex |
|  |  | Strychnine | Strychni semen |
| 6 | TAS2R8 | Parthenolide | Tanacetum vulgare |
|  |  |  |  |
| 7 | TAS2R9 | - |  |
| 8 | TAS2R10 | Absinthin | Artemisia absinthum herba |
|  |  | Arborescin | Artemisia myriantha |
|  |  | Arglabin | Artemisia myriantha |
|  |  | Artemorin | Laurus nobilis folium |
|  |  | Benzoin | Styrax tonkinensis |
|  |  | Caffeine | Camellia sinensis folium |
|  |  | Camphor | Cinnamomum camphora rhadix |
|  |  | Cascarillin | Rhamnus purshiana cortex |
|  |  | Costunolide | Aucklandia lappa Decne, Vladimiriae Radix |
|  |  | Coumarin | Artemisiae argyi folium, Cinnamomi ramulus, Carotae fructus |
|  |  | Cucurbitacin B | Luffae fructus retinervus |
|  |  | Cucurbitacin E | Bolbostemmatis rhizoma |
|  |  | Papaverine | Papaveris pericarpium |
|  |  | Parthenolide | Tanacetum vulgare |
|  |  | Picrotoxinin | Anamirta cocculus fructus,  Artemisiae argyi folium |
|  |  | Quassin | Picrasmae ramulus et folium |
|  |  | Quinine | Cinchona ledgeriana cortex |
|  |  | Strychnine | Strychni semen |
|  |  | (-)-α-Thujon | Platycladi Cacumen, Artemisiae argyi folium |
|  |  |  |  |
|  |  |  |  |
| 9 | TAS2R13 | - |  |
| 10 | TAS2R14 | Absinthin | Artemisia absinthum herba |
|  |  | Apigenin | Ginkgo semen, Siphonostegiae herba, |
|  |  | Arborescin | Artemisia myriantha |
|  |  | Arglabin | Artemisia myriantha |
|  |  | Aristolochic acid | Aristolochiae fructus |
|  |  | Artemorin | Laurus nobilis folium |
|  |  | Azathioprine | Azathioprine |
|  |  | Benzoin | Styrax tonkinensis |
|  |  | Caffeine | Camellia sinensis folium |
|  |  | Camphor | Cinnamomum Camphora rhadix |
|  |  | Cascarillin | Rhamnus purshiana cortex |
|  |  | Coumarin | Artemisiae argyi folium, Cinnamomi ramulus, Carotae fructus |
|  |  | Costunolide | Aucklandia lappa decne, Vladimiriae radix |
|  |  | Cucurbitacin B | Luffae fructus retinervus |
|  |  | Datiscetin | Rubia cordifolia rhizoma |
|  |  | Eriodictyol | Scutellariae radix |
|  |  | Falcarindiol | Saposhnikoviae divaricatae radix, Daucus carota L |
|  |  | Flavone | Andrographis herba |
|  |  | Genistein | Puerariae radix,  Sojae atricolor semen,  Flos sophorae |
|  |  | Homoeriodictyol | Viscum coloratum (Kom.) Nakai |
|  |  | Humulone isomers | Humulus lupulus |
|  |  | Liquiritigenin | Glycyrrhizae radix,  Desmodii styracifolii herba,  Lignum dalbergiae odoriferae rosewood |
|  |  | Luteolin | Siphonostegiae herba,  Cirsii japonici herba |
|  |  | Kaempferol | Anisi stellati fructus, Ginkgo semen |
|  |  | 6-Methoxyluteolin | Flos lonicerae, |
|  |  | Naringenin | Desmodii styracifolii herba, Folium artemisiae argyi |
|  |  | Papaverine | Papaveris pericarpium |
|  |  | Parthenolide | Tanacetum vulgare |
|  |  | Picrotoxinin | Anamirta cocculus fructus,  Artemisiae argyi folium |
|  |  | Pinocembrin | Alpiniae katsumadai semen,  Curcumae rhizoma,  Glycyrrhizae radix |
|  |  | Quassin | Picrasmae ramulus et folium |
|  |  | Quercetin | Artemisiae argyi folium, Ginkgo semen, Glehniae radix, Sophorae flos, Polygoni cuspidati rhizoma et radix, Rhododendri daurici, Taxilli herba, Agrimoniae herba, Typhae pollen folium |
|  |  | Quinine | Cinchona ledgeriana cortex |
|  |  | Resveratrol | Polygoni multiflori radix ,  Polygoni cuspidati rhizoma |
|  |  | Scutellarein | Herba scutellariae barbatae,  Orozylum indicum |
|  |  | Silibinin | Silybum marianum |
|  |  | (-)-α-Thujon | Platycladi cacumen, Artemisiae argyi folium |
|  |  | 5,7,2′-Trihydroxyflavone | Scutellariae radix |
| 11 | TAS2R16 | Sinigrin | Isatidis radix, Sinapis albae semen, Thlaspi arvense L |
| 12 | TAS2R19 | - |  |
| 13 | TAS2R20 | - |  |
| 14 | TAS2R30 | Absinthin | Artemisia absinthum herba |
|  |  | Amarogentin | Cynanchi stauntonii rhizoma et radix, Swertiae herba,  Gentiana manshurica kitag |
|  |  | Andrographolide | Andrographis herba |
|  |  | Artemorin | Laurus nobilis folium |
|  |  | Camphor | Cinnamomum camphora rhadix |
|  |  | Cascarillin | Rhamnus purshiana cortex |
|  |  | Picrotoxinin | Anamirta cocculus fructus,  Artemisiae argyi folium |
|  |  | Quassin | Picrasmae ramulus et folium |
| 15 | TAS2R31 | Parthenolide | Tanacetum vulgare |
|  |  | Quinine | Cinchona ledgeriana cortex |
| 16 | TAS2R38 | Limonin | Atractylodis rhizome, Rhizoma coptidis,  Fructus aurantii immaturus |
|  |  | Sinigrin | Isatidis Radix,  Sinapis albae semen,  Thlaspi arvense L |
| 16 | TAS2R39 | Apigenin | Ginkgo semen, Siphonostegiae herba, |
|  |  |  |  |
|  |  | Amarogentin | Cynanchi stauntonii rhizoma et radix,  Swertiae herba,  Gentiana manshurica kitag |
|  |  | Colchicine | Lilii bulbus |
|  |  | 3,2′-Dihydroxychalcone | Caulis ephedrae/ephedra |
|  |  | (-)-Epicatechin (EC) | Ginkgo semen, Rhei radix et rhizoma |
|  |  | Eriodictyol | Astragalus chrysopterus bunge |
|  |  | Flavone | Andrographis herba |
|  |  | Genistein | Puerariae radix,  Semen sojae atricolor,  Flos Sophorae |
|  |  | Gossypetin | Folium rhododendri daurici |
|  |  | Homoeriodictyol | Viscum coloratum (Kom.) Nakai |
|  |  | 7-Hydroxyisoflavone | Puerariae radix |
|  |  | Liquiritigenin | Glycyrrhizae radix,  Desmodii styracifolii herba,  Lignum dalbergiae odoriferae rosewood |
|  |  | Luteolin | Siphonostegiae herba，  Cirsii japonici herba |
|  |  | Kaempferol | Anisi stellati fructus, Ginkgo semen |
|  |  | 6-Methoxyluteolin | Flos lonicerae, |
|  |  | Naringenin | Desmodii styracifolii herba, Folium artemisiae argyi |
|  |  | Phloretin | Appple pericarp |
|  |  | Pinocembrin | Alpiniae katsumadai semen,  Curcumae rhizoma,  Glycyrrhizae radix |
|  |  | Quinine | Cinchona ledgeriana cortex |
|  |  | Resveratrol | Polygoni multiflori radix ,  Polygoni cuspidati rhizoma |
|  |  | Scutellarein | Scutellariae barbatae herba,  Orozylum indicum |
|  |  | Silibinin | Silybum marianum |
|  |  | Theaflavin (TF1) | Camellia sinensis  folium |
|  |  | Theaflavin-3’-*O*-gallate (TF2B) | Camellia sinensis  folium |
|  |  | Theaflavin-3,3’-*O*-digallate (TF3) | Camellia sinensis  folium |
|  |  | Thiamine | Allii sativi bulbus |
|  |  | 5,7,2′-Trihydroxyflavone | Ferulae cha resina |
|  |  | 7,3′,4′-Trihydroxyflavone | Pyrola atropurpurea franch |
|  |  | 7,8,4′-Trihydroxyisoflavone | Pyrola atropurpurea franch,  Crotonis fructus,  Fructus momordicae |
| 18 | TAS2R40 | Humulone isomers | Humulus lupulus |
|  |  | Quinine | Cinchona ledgeriana cortex |
| 19 | TAS2R41 | - |  |
| 20 | TAS2R42 | - |  |
| 21 | TAS2R43 | Aloin | Fructus hordei germinatus |
|  |  | Amarogentin | Cynanchi stauntonii rhizoma et radix,  Swertiae herba,  Gentiana manshurica kitag |
|  |  | Arborescin | Artemisia myriantha |
|  |  | Arglabin | Artemisia myriantha |
|  |  | Aristolochic acid | Aristolochiae fructus |
|  |  | Caffeine | Camellia sinensis folium |
|  |  | Falcarindiol | Saposhnikoviae divaricatae radix, Daucus carota L |
|  |  | Grossheimin | Cynara scolymus |
|  |  | Helicin | Fitipenduta ulmaria herba |
|  |  | Quinine | Cinchona ledgeriana cortex |
| 22 | TAS2R45 | - |  |
| 23 | TAS2R46 | Absinthin | Artemisia absinthum herba |
|  |  | Amarogentin | Cynanchi stauntonii rhizoma et radix, Swertiae herba,  Gentiana manshurica kitag |
|  |  | Andrographolide | Andrographis herba |
|  |  | Arborescin | Artemisia myriantha |
|  |  | Arglabin | Artemisia myriantha |
|  |  | Artemorin | Laurus nobilis folium |
|  |  | Brucine | Strychni semen |
|  |  | Caffeine | Camellia sinensis folium |
|  |  | Cascarillin | Rhamnus purshiana cortex |
|  |  | Cnicin | Cnicus benedictus |
|  |  | Colchicine | Lilii bulbus |
|  |  | Costunolide | Aucklandia lappa decne, Vladimiriae radix |
|  |  | Grossheimin | Cynara scolymus |
|  |  | Parthenolide | Tanacetum vulgare |
|  |  | Penten-3-ol | Pericarpium zanthoxyli |
|  |  | Picrotoxinin | Anamirta cocculus fructus,  Artemisiae argyi folium |
|  |  | Quassin | Picrasmae ramulus et folium |
|  |  | Quinine | Cinchona ledgeriana cortex |
|  |  | Strychnine | Strychni semen,  Cynanchi stauntonii Rhizoma et radix,  Portulacae herba |
|  |  |  |  |
| 24 | TAS2R50 | Amarogentin | Cynanchi stauntonii rhizoma et radix,  Swertiae herba,  Gentiana manshurica kitag |
|  |  |  |  |
|  |  |  |  |
|  |  | Andrographolide | Andrographis herba |
| 25 | TAS2R60 | - |  |

Note: ‘-’ means no correlated bitter agonists from TCM. According to the bitter database built in 2012 (http://bitterdb.agri.huji.ac.il/dbbitter.php), a compilation of 25 hTAS2Rs with or without bitter compounds from bitter flavors is depicted in Table S1. However, more compounds from bitter flavors than those listed have been identified including terpenes, diterpenes, triterpenes, sesquiterpene lactones, flavones, bitter acids of the humulone series, and glycosides.

**Table S2 Bitter flavors with hemostatic, blood-activating, and stasis-dispelling functions ([**[**1**](#_ENREF_1)**;** [**2**](#_ENREF_2)**; 3])**

| No. | Degree of bitterness | TCM | Herb | Component |
| --- | --- | --- | --- | --- |
| 1 | ++ | Corydalis decumbentis rhizoma | Corydalis decumbens | Protopine, Palmatine chloride, 8-(5,6,7,8-Tetrahydro-6-methyl-1,3-dioxolo[4,5-g]isoquinolin-5-yl)furo[3,4-e]-1,3-benzodioxol-6(8H)-one, (+)-Corlumidine |
| 2 | ++ | Corydalis rhizoma | Corydalis yanhusuo | Isoquinoline type alkaloid, Tetrahydropalmatine, Hydrochloride |
| 3 | + | Croci stigma | Crocus sativus | Crocin,(4S)-2,6,6-trimethyl-4-[(2R,3R,4S,5S,6R)-3,4,5-trihydroxy-6-(hydroxymethyl)oxan-2-yl]oxy-cyclohexene-1-carbaldehyde |
| 4 | ++ | Dioscoreae nipponicae rhizoma | Dioscorea nipponica | Dioscin, Gracillin, Asperin, 25-D-spirosta-3,5-diene, Piscidic acid, Steroidal saponins, Dioscin |
| 5 | + | Erigerontis herba | Erigeron breviscapus | Scutellarin (TAS2R14, 39), Isoflavones |
| 6 | + | Ginkgo folium | Ginkgo biloba | (-)-Epicatechin (EC) (TAS2R4, 5, 39), Kaempferol (TAS2R14, 39), Ginkgetin, Ginkgolide (*), Quercetin dehydrate, Isorhamnetin |
| 7 | + | Leonurus japonicus | Leonuri japonicus | Stachysite, Leonurine, N,N-Dimethyl-L-prolin |
| 8 | ++ | Chuanxiong rhizoma | Ligusticum chuanxiong | Chuanxiongol, Ferulic acid, 4-hydroxy- 3- butylphthalide, Senkyunolide, Ligustilide, Tetramethylpyrazine |
| 9 | + | Moutan cortex | Paeonia suffruticosa | Pentagalloylglucose (PGG) (TAS2R5, TAS2R39), Paeonol |
| 10 | + | Persicae semen | Prunus persica | Amygdalin (TAS2R16) |
| 11 | + | Rabdosiae rubescentis herba | Rabdosia rubescens | Oridonin |
| 12 | ++ | Salviae Miltiorrhizae radix et rhizoma | Salviae Miltiorrhizae | Salvianolic acids, Tanshinone |
| 13 | + | Saussureae Involucratae herba | Saussurea involucrata | Rutin, alkaloid |
| 14 | + | Vaccariae semen | Vaccaria segetalis | Vacsegoside, Vaccarin |
| 15 | ++ | Verbenae herba | Verbena officinatis L. | Cornin, Oleanic acid, Ursolic acid , β-sitosterol |
| 16 | + | Angelicae sinensis radix | Angelica sinensis | Ferulic acid, Ligusiilide, N-butylidene phthalide, Nicotinic acid |
| 17 | + | Arnebiae radix | Arnebia euchroma | Shikonin |
| 18 | + | Aconiti radix | Aconitum carmichaelii | Aconitine, Hypaconitine, Mesaconitine |
| 19 | + | Potentillae chinensis herba | Potentilla chinensis | Fumaric acid, Gallic acid, 3,4-Dihydroxybenzoic acid, Kaempferol (TAS2R14, 39), Naringenin (TAS2R14), Quercetin dihydrate, |
| 20 | ++ | Notoginseng radix ex rhizoma | Panax notoginseng | Ginsengrootextract, Notoginsenoside R1 |
| 21 | ++ | Curccumae longae rhizoma | Curccuma longa | Benzine, Curcumin, Sesquiterpineol, Turmeric P.E, Turmerone, (1S,4R,6R)-1,7,7-Trimethylbicyclo[2.2.1]heptan-6-ol |
| 22 | + | Raphani semen | Raphanus sativus | Sinapine, Raphanin |
| 23 | ++ | Auranti fructus | Citrus aurantium | Aurantiamrin, Aurantiamaric acid, Hesperidin, Limonin (TAS2R38), Naringin (*), Neohesperidin |
| 24 | ++ | Liriopes radix | Liriope spicata | Liriopesides, Ophiopogonin D, Ursolic acid, Vanillic acid |
| 25 | + | Acanthopanacis Senticosi radix et rhizoma seu caulis | Acanthopanax seuticosus | Eleutheroside |
| 26 | + | Trichosanthis Fructus | Trichosanthis fructus | Resin, 2.3.5-Triphenyl tetrazolium chloride |
| 27 | ++ | Nelumbinis plumula | Nelumbo nucifera | Liensinine, Neferine, Nuciferine, Isoliensinine |
| 28 | + | Ophiopogonis radix | Ophiopogon japonicus | Alkonoid, Steroidal saponins, Zbeta-Sitosterol |
| 29 | ++ | Periplocae cortex | Periploca sepium | Peripolocymarin, 2-Hydroxy-4-methoxybenzaldehyde, β-sitosterol |
| 30 | ++ | Polygalae radix | Polygala tenuifolia | Tenuifolin, 3, 6′-Disinapoyl Sucrose |
| 31 | + | Rhodiolae crenulatae *radix et rhizoma* | Rhodiola crenulata | Salidroside |
| 32 | + | Taxilli herba | Taxillus chinensis | Avicularin, Cardenolide, Quercetin (TAS2R14) |

Note: +, ++ represents slight bitter, bitter, respectively, recorded in *The Pharmacopoeia of the People's Republic of China 2015 Edition* . * means this compound is bitter with unknown correlated TAS2Rs.

**Table S3 Bitter flavors with cough-suppressing and panting-calming functions ([**[**1**](#_ENREF_1)**;** [**2**](#_ENREF_2)**; 3])**

| No. | Degree of bitterness | TCM | Herb | Component |
| --- | --- | --- | --- | --- |
| 1 | ++ | Artemisiae argyi folium | Artemisia argyi | α-thujone (TAS2R10, 14), α-phellandrene, β-caryophyllene, Absinthin (TAS2R10, 14, 46, 47), Camphene, Camphor (TAS2R4, 10, 14, 47), Carvone, |
| 2 | + | Aucklandiae radix | Aucklandia lappa Decne | Alantolactone, Isoalantolactone |
| 3 | ++ | Aquilariae lignum resinatum | Aquilaria sinensis | Agarotetrol |
| 4 | + | Ardisiae japonicae herba | Ardisia japonica | Ardisin, Ardisinol, Flavonoid glycosides, Dergenin, Embelin, Rapanone |
| 5 | + | Benzoinum | Styrax tonkinensis | Benzoin (TAS2R10, TAS2R14) |
| 6 | ++ | Catechu | Aazck catechu | Catechin hydrate, Epicatechin (TAS2R4, 5, 39) |
| 7 | + | Chelidonii herba | Chelidonium majus | α-，β-homochelidonine, Berberine (*), Coptisine chloride, Chelerythrine chloride, Chelidonine, Sanguinarine, Protopine |
| 8 | + | Genkwa flos | Daphne genkwa | Apigenin (TAS2R14, 39), Beta-Sitosterol, Hydroxygenkwanin |
| 9 | + | Descurainia semen | Descurainia sophia | Sinigrin (TAS2R16, 38) |
| 10 | ++ | Dioscoreae nipponicae rhizoma | Dioscorea nipponica | Asperin, Dioscin, Gracillin, Piscidic acid, Steroidal saponins, 25-D-spirosta-3,5-diene |
| 11 | + | Ephedrae herba | Ephedra sinica | Ephedrine hydrochloride, Pseudoephedrine hydrochloride, 3,2′-Dihydroxychalcone (TAS2R14, 39) |
| 12 | + | Eriobotryae folium | Eriobotrya japonical | Oleanic acid, Ursolic acid |
| 13 | ++ | Eupatorii lindleyanum | Eupatorii lindleyani herba | Hyperoside |
| 14 | + | Farfarae flos | Tussilago farfara | Rutin, Rutoside, Tussilagone |
| 15 | + | Kansui radix | Euphorbia kansui | Kansuinin A, Kansuinine B |
| 16 | + | Ginkgo Semen | Ginkgo biloba | Amentoflavone, Bilobetin, (-)-Epicatechin (EC) (TAS2R4, 5, 39) , Ginkgetin, Ginkgolide(*), Kaempferol (TAS2R14, 39), Quercetin dihydrate, Rutin, |
| 17 | + | Ginkgo folium | Ginkgo biloba | (-)-Epicatechin (EC) (TAS2R4, 5, 39), Kaempferol (TAS2R14, 39), Ginkgetin, Ginkgolide (*), Quercetin dehydrate, Isorhamnetin |
| 18 | + | Ginseng radix et rhizoma |  | Ginsengrootextract |
| 19 | + | Hyoscyami semen | Hyoscyamus niger | L-Hyoscyamine, Scopolamine |
| 20 | + | Inula flos | Inula japonica | β-sitosterol, Quercetin (TAS2R14), Caffeic Acid (*), Kaempferol (TAS2R14, 39), |
| 21 | + | Iridis tectori rhizoma | Iris tectorum | Tectoridin |
| 22 | + | Linderae radix | Lindera aggregata | Laurolitsine, Liderane |
| 23 | ++ | Liriopes radix | Liriope spicata | Liriopesides, Ophiopogonin D, Ursolic acid, Vanillic acid |
| 24 | + | Magnoliae officinalis cortex | Magnolia officinalis | Magnolol, Magnocurarine |
| 25 | ++ | Myrrha | Commiphora myrrha | Amberlite, Artemisia terpenoids, Benzine, Cinnasaldehyde, Cadinene, Commiphoric acid, Formic acid, Limonene, Magnesium oxide, Myrrholic acid, Ruthenium acetate |
| 26 | + | Perilla fructs | Perilla frutescens | Rosmarinic acid |
| 27 | + | Peucedani decursivi | Peucedanum decursivum | Nodakenin |
| 28 | + | Peucedani radix | Peucedanum praeruptorum | Praeruptorin B |
| 29 | + | Pharbitidis semen | Pharbitis nil | α-methylbutyric acid, Nilic acid, Pharbitin, Pharbitic acid, Tiglic acid, Valeric acid |
| 30 | + | Physochlainae radix | Physochlaina infundibularis | L-Hyoscyamine |
| 31 | + | Pinelliae Rhizoma | Pinellia ternata | Succinic acid |
| 32 | + | Polygoni tinctorii folium | Polygonum tinctorium | Indigo |
| 33 | ++ | Armeniacae semen amarum | Prunus armeniaca | ﻿Amygdalin (TAS2R16) |
| 34 | + | Persicae semen | Prunus persica | ﻿Amygdalin (TAS2R16), Beta-Glucosidase, Glyceryl Monooleate, |
| 35 | + | Pyrrosiae folium | Pyrrosia sheareri | β-sitosterol, Anthraquinone, Chlorogenic acid, Diploptene, Flavone (TAS2R14, 39), Saponin |
| 36 | + | Rhodiolae crenulatae radix et rhizoma | Rhodiola crenulata | Salidroside |
| 37 | ++ | Rhododendri daurici folium | Rhododendron dauricum | Farrerol, Gossypetin (TAS2R39), Tannic acid |
| 38 | ++ | Seringae cortex | Seringa reticulata | Syringin |
| 39 | + | Suis fellis pulvis | Sus scrofadomestica | Dehydrocholic acid (*), Taurocholic acid (TAS2R4) |
| 40 | + | Viticis negundo folium | Vitex negundo | β-caryophyllene, Faradiol, Hyperoside, Rutin, Tannic acid, |

Note: +, ++ represents slight bitter, bitter, respectively, recorded in *The Pharmacopoeia of the People's Republic of China 2015 Edition* . * means this compound is bitter with unknown correlated TAS2Rs.

**Table S4 Bitter flavors mainly used for the treatment of diseases in gastrointestinal tract ([**[**1**](#_ENREF_1)**;** [**2**](#_ENREF_2)**; 3])**

| No. | Degree of bitterness | | TCM | Herb | Component |
| --- | --- | --- | --- | --- | --- |
| 1 | + | Arecae semen | | Areca catechu | Arecoline hydrobromide (*) |
|  | + | Atractylodis Macrocephalae Rhizoma | | Atractylodis macrocephalae | Atractylenolide-1, Atractylol, Atractylon |
|  | + | Atractylodis rhizoma | | Atractylodes lancea | 2-carene, chamigrene, chamigrene, 3β-acetoxyatractylone, 3β-hydroxyatracetylone, butenoliede |
|  |  | Aucklandiae radix | | Aucklandia lappa | Costunolide (TAS2R10, 14, 46), Dehydrocostuslactone |
|  | ++ | Auranti fructus | | Citrus aurantium | Aurantiamrin, Aurantiamaric acid, Hesperidin, Limonin (TAS2R38), Naringin (*), Neohesperidin (*) |
|  | ++ | Carotae fructus | | Daucus carota | Asarone, Biasabolene, Tiglic acid |
| 2 | ++ | Chuanxiong rhizoma | | Ligusticum chuanxiong | Chuanxiongol, Ferulic acid, Ligustilide, Senkyunolide, Tetramethylpyrazine, 4-hydroxy- 3- butylphthalide |
|  | + | Citri fructus | | Citrus medica | Naringin (*), Neohesperidin (*) |
| 3 | ++ | Citri grandis exocapium | | Citrus grandis | Citral, Geraniol, Linalool, Naringenin (TAS2R14), Naringin (*) |
| 4 | + | Citri reticulatae pericarpium | | Citrus reticulata | Limonin (TAS2R38), Limonolid, Naringin (*), Quassin (TAS2R 4, 10, 14, 46), Neohesperidin (*) |
|  | + | Citri sarcodactylis fructus | | Citrus medica | Limonin (TAS2R38), Hesperidin |
|  | + | Curcuma rhizoma | | Curcuma phaeocaulis | Scutellarein (TAS2R14, 39), Aerugidiol, Ar-turmerone, Borneo1, Caryophyllene epoxide, Caryophyllene epoxide, Camphene, Caryophyllene, Curzenone, Curcurmenol, Curcumene, Cudione, Difurocumenone, Pormacrone, Pinene, Limonene, 1,8-Cineole, Terpinen, Isborneol, Isourecumenol, Turme |
| 5 | - | Dianthi herba | | Dianthus superbus | Isoorientin |
| 6 | ++ | Euodiae fructus | | Euodia rutaecarpa | Evodiamine, Limonin (TAS2R38), Rutaecarpine, Rutaevin |
| 7 | + | Ferulae resina | | Ferula sinkiangensis | Alpha-pinene, 5,7,2′-Trihydroxyflavone (TAS2R39), |
| 8 | ++ | Ilicis rotundae cortex | | Ilex rotunda | Eleutheroside B, Pedunculoside |
| 9 | ++ | Inulae radix | | Inula helenium | Alantolactone, Isoalantolactone |
| 10 | - | Kaempferiae rhizoma | | Kaempferia galanga L | Ethyl-p-methoxycinnamate，Kaempferol (TAS2R14, 39), Kaempferitrin |
| 11 | + | Kansui radix | | Euphorbia kansui | Kansuinin A, Kansuinine B |
| 12 | + | Magnoliae officinalis cortex | | Magnolia officinalis | Magnolol, Magnocurarine |
| 13 | + | Myristicae semen | | Myristica fragrans | Bornylene, Limonene, Sabinene, Terpinen-4ol,γ-Terpinene |
| 14 | ++ | Myrrha | | Commiphora myrrha | Amberlite, Artemisia terpenoids, Benzine, Cadinene, Cinnasaldehyde, Commiphoric acid, Formic acid, Magnesium oxide, Limonene, Myrrholic acid, Ruthenium acetate |
| 15 | + | Papaveris pericarpium | | Papaver somniferum | Morphine, Papaverine (TAS2R7, 10, 14) |
| 16 | + | Pharbitidis semen | | Pharbitis nil | α-methylbutyric acid, Nilic acid, Pharbitin, Pharbitic acid, Tiglic acid, Valeric acid |
| 17 | + | Polygoni avicularis herba | | Polygonum aviculare | Avivcularin, Hyperoside, Hyperoside, Isovitexin, Luteolin (TAS2R14, 39), Myricitrin, Quercetin (TAS2R14), Quercitrin, Rhamnetin-3-galactoside, Vitexin |
| 18 | + | Pruni semen | | Prunus humilis | Amygdalin (TAS2R16), Sennoside |
| 19 | + | Pruni semen | | Prunus humilis | Amygdalin(TAS2R16), Sennoside |
| 20 | + | Raphani semen | | Raphanus sativus | Raphanin, Sinapine |
| 21 | ++ | Rhei radix et rhizoma | | Rheum officinale Bail | Aloe emodin, Chrysophanic acid, Emodin, Emodin-3-methyl ethe, (-)-Epicatechin (EC) (TAS2R4, 5, 39), Flavone (TAS2R14, 39), Rhein, Sennoside, |
| 22 | + | Sennae folium | | Cassia angustifolia | Kaempferol (TAS2R14, 39), Physcion, Chrysophanol, Rhein, Sennoside A, Sennoside C, Sennoside B, Aloeemodin, Bianthrone, Sennoside D |
| 23 | + | Sparganii rhizoma | | Sparganiuum stoloniferum | Benzeneethanol, Dehydrocostuslactone, Hexadecanoic acid, 1,4-benzenedio, |
| 24 | + | Typhae pollen | | Typha angustifolia | Isorhamnetin-3-O-neohespeidoside, Quercetin (TAS2R14), Typhaneoside |
| 25 | ++ | Vladimiriae radix | | Vladimiria souliei | Costunolide (TAS2R10, 14, 46), Dehydrocostuslactone |

Note: +, ++ represents slight bitter, bitter, respectively, recorded in *The Pharmacopoeia of the People's Republic of China 2015 Edition* . * means this compound is bitter with unknown correlated TAS2Rs.

**Table S5 Bitter flavors mainly used for tocolytics or promoting labor ([**[**1**](#_ENREF_1)**;** [**2**](#_ENREF_2)**; 3])**

| No. | Degree of bitterness | TCM | Herb | Component |
| --- | --- | --- | --- | --- |
| 1 | ++ | Atractylodis macrocephalae rhizoma | Atractylodis macrocephalae | Atractylenolide (*), Atractyloside potassium salt, Limonin (TAS2R38), |
| 2 | ++ | Inulae radix | Inula helenium | Alantolactone, Isoalantolactone |
| 3 | + | Leonuri herba | Leonuri japonicus | N,N-Dimethyl-L-prolin , Leonurine, Stachysite |
| 4 | ++ | Scutellariae radix | Scutellaria baicalensis | Baicailin, Baicalein, Scutellarein (TAS2R14, 39) |
| 5 | + | Taxilli herba | Taxillus chinensis | Avicularin, Cardenolide, Quercetin (TAS2R14), |
| 6 | ++ | Verbenae herba | Verbena officinatis | β-sitosterol, Cornin, Oleanic acid, Ursolic acid |
| 7 | + | Visci herba | Viscum coloratum | β-amyrin, Flavonoids, Homoeriodictyol (TAS2R14, 39), Oleanolicacid (*)， Lupeol,3-o-(p-D-glucopyranosy1)-oleanolicacid (*), Mesoinositol |

Note: +, ++ represents slight bitter, bitter, respectively, recorded in *The Pharmacopoeia of the People's Republic of China 2015 Edition* . * means this compound is bitter with unknown correlated TAS2Rs.

**References:**

[1] A. Jaggupilli, R. Howard, J.D. Upadhyaya, R.P. Bhullar, and P. Chelikani, Bitter taste receptors: Novel insights into the biochemistry and pharmacology. The International Journal of Biochemistry & Cell Biology 77, Part B (2016) 184-196.

[2] Pharmacopoeia Committee of the People’s Republic of China, Pharmacopoeia Committee of the People’s Republic of China 2015 Edition, Chinese Medical Science and Technology Press, 2015.

[3] A. Dagan-Wiener, A. Di Pizio, I. Nissim, M.S. Bahia, N. Dubovski, E. Margulis, and M.Y. Niv, BitterDB: taste ligands and receptors database in 2019. Nucleic Acids Research 47 (2019) D1179-D1185.
